# Ethyl 2-benzoyl-6-methyl­indolizine-7-carboxyl­ate

**DOI:** 10.1107/S1600536812016212

**Published:** 2012-04-21

**Authors:** Shang-Tie Liao

**Affiliations:** a School of New Energy Science and Engineering, Xinyu University, Xinyu 338000, People’s Republic of China, and, Key Laboratory of Jiangxi University for Silicon Materials, Xinyu 338000, People’s Republic of China

## Abstract

The title compound, C_19_H_17_NO_3_, was synthesized using a tandem annulation reaction between 4-benzoyl-1*H*-pyrrole-2-carbaldehyde and (*E*)-ethyl 4-bromo­but-2-enoate under mild conditions. The dihedral angle between the benzene ring and the indolizine ring system is 41.73 (4)°.

## Related literature

For background to indolizines, see:Ge *et al.* (2009*a* ▶, 2011 ▶). For bond lengths and angles in related structures, see: Ge *et al.* (2009*b* ▶). For the synthesis of imidazo[1,2-*a*]pyridines *via* a tandem reaction, see: Jia *et al.* (2010 ▶).
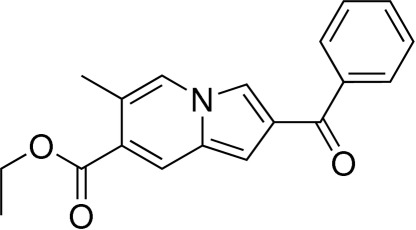


## Experimental

### 

#### Crystal data

C_19_H_17_NO_3_
*M* *_r_* = 307.34
Monoclinic, 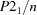

*a* = 8.177 (5) Å
*b* = 17.243 (5) Å
*c* = 11.191 (5) Å
β = 102.070 (5)°
*V* = 1543.0 (13) Å^3^
*Z* = 4
Mo *K*α radiation
μ = 0.09 mm^−1^
*T* = 293 K
0.18 × 0.15 × 0.14 mm

#### Data collection

Bruker SMART APEX CCD area-detector diffractometer
Absorption correction: multi-scan (*SADABS*; Bruker, 2005 ▶) *T*_min_ = 0.983, *T*_max_ = 0.990
8583 measured reflections
3150 independent reflections
2434 reflections with *I* > 2σ(*I*)
*R*_int_ = 0.124

#### Refinement

*R*[*F* ^2^ > 2σ(*F* ^2^)] = 0.056
*wR*(*F* ^2^) = 0.166
*S* = 1.04
3150 reflections
211 parameters
H-atom parameters constrained
Δρ_max_ = 0.30 e Å^−3^
Δρ_min_ = −0.22 e Å^−3^

### 

Data collection: *APEX2* (Bruker, 2005 ▶); cell refinement: *SAINT* (Bruker, 2005 ▶); data reduction: *SAINT*; program(s) used to solve structure: *SHELXS97* (Sheldrick, 2008 ▶); program(s) used to refine structure: *SHELXL97* (Sheldrick, 2008 ▶); molecular graphics: *XP* in *SHELXTL* (Sheldrick, 2008 ▶); software used to prepare material for publication: *SHELXL97*.

## Supplementary Material

Crystal structure: contains datablock(s) I, global. DOI: 10.1107/S1600536812016212/hg5204sup1.cif

Structure factors: contains datablock(s) I. DOI: 10.1107/S1600536812016212/hg5204Isup2.hkl

Supplementary material file. DOI: 10.1107/S1600536812016212/hg5204Isup3.cml

Additional supplementary materials:  crystallographic information; 3D view; checkCIF report

## Figures and Tables

**Fig. 1.**
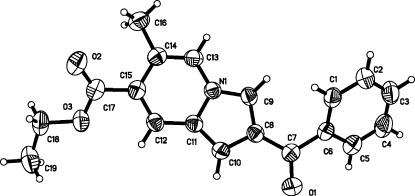
The molecular structure of (I), showing displacement ellipsoids drawn at the 50% probability level.

## supplementary crystallographic information

### Comment

Indolizines have attracted considerable attention from medicinal and organic chemists because of the interesting similarities and diversions in structure to indole (Ge *et al.*; 2009*a*, 2011). Synthetic indolizines play important roles as calcium entry blockers, potential central nervous system depressants, 5-HT3 receptor antagonist, histamine H3 receptor antagonists, cardiovascular agents, and PLA2 inhibitors. They have also drawn much attention owing to their possible usage as dyes and chemosensors. The title indolizine (I) (Fig. 1) was synthesized in order to study its biological properties. (I) was screened for anticancer activities and found to be inactive. We report here the crystal structure of the title compound. In the title compound, C_19_H_17_NO_3_, all bond lengths and angles show normal values (Ge *et al.*, 2009*b*). The dihedral angle between the benzene and indolizine rings is 41.73 (4)°.

### Experimental

To a 50 ml round-bottomed flask were added 4-benzoyl-1*H*-pyrrole-2-carbaldehyde (1.00 mmol), (*E*)-ethyl 4-bromobut-2-enoate (2.00 mmol), potassium carbonate (0.28 g, 2.05 mmol) and dry DMF (10 ml). The mixture was stirred at room temperature for 8 h. The solvent was removed under reduced pressure and an product was isolated by column chromatography on silica gel (yield 76%). Crystals of (I) suitable for X-ray diffraction were obtained by allowing a refluxed solution of the product in ethyl acetate to cool slowly to room temperature (without temperature control) and allowing the solvent to evaporate for 10 d.

### Refinement

All H atoms were placed in geometrically calculated positions and refined using a riding model with C—H = 0.97 Å (for CH_2_ groups),0.96 Å (for CH_3_ groups) and 0.93 Å (for aromatic protons), their isotropic displacement parameters were set to 1.2 times (1.5 times for CH_3_ groups) the equivalent displacement parameter of their parent atoms.

### Crystal data

**Table:**

C_19_H_17_NO_3_	*F*(000) = 648
*M**_r_* = 307.34	*D*_x_ = 1.323 Mg m^−^^3^
Monoclinic, *P*2_1_/*n*	Mo *K*α radiation, λ = 0.71069 Å
Hall symbol: -P 2yn	Cell parameters from 4026 reflections
*a* = 8.177 (5) Å	θ = 2.4–28.4°
*b* = 17.243 (5) Å	µ = 0.09 mm^−^^1^
*c* = 11.191 (5) Å	*T* = 293 K
β = 102.070 (5)°	Block, yellow
*V* = 1543.0 (13) Å^3^	0.18 × 0.15 × 0.14 mm
*Z* = 4	

### Data collection

**Table:**

Bruker SMART APEX CCD area-detector diffractometer	3150 independent reflections
Radiation source: fine-focus sealed tube	2434 reflections with *I* > 2σ(*I*)
Graphite monochromator	*R*_int_ = 0.124
phi and ω scans	θ_max_ = 26.4°, θ_min_ = 2.2°
Absorption correction: multi-scan (*SADABS*; Bruker, 2005)	*h* = −10→10
*T*_min_ = 0.983, *T*_max_ = 0.990	*k* = −17→21
8583 measured reflections	*l* = −13→9

### Refinement

**Table:**

Refinement on *F*^2^	Secondary atom site location: difference Fourier map
Least-squares matrix: full	Hydrogen site location: inferred from neighbouring sites
*R*[*F*^2^ > 2σ(*F*^2^)] = 0.056	H-atom parameters constrained
*wR*(*F*^2^) = 0.166	*w* = 1/[σ^2^(*F*_o_^2^) + (0.0781*P*)^2^ + 0.2178*P*] where *P* = (*F*_o_^2^ + 2*F*_c_^2^)/3
*S* = 1.04	(Δ/σ)_max_ < 0.001
3150 reflections	Δρ_max_ = 0.30 e Å^−^^3^
211 parameters	Δρ_min_ = −0.22 e Å^−^^3^
0 restraints	Extinction correction: *SHELXL97* (Sheldrick, 2008), Fc^*^=kFc[1+0.001xFc^2^λ^3^/sin(2θ)]^-1/4^
Primary atom site location: structure-invariant direct methods	Extinction coefficient: 0.023 (4)

### Special details

**Table:**

Geometry. All e.s.d.'s (except the e.s.d. in the dihedral angle between two l.s. planes) are estimated using the full covariance matrix. The cell e.s.d.'s are taken into account individually in the estimation of e.s.d.'s in distances, angles and torsion angles; correlations between e.s.d.'s in cell parameters are only used when they are defined by crystal symmetry. An approximate (isotropic) treatment of cell e.s.d.'s is used for estimating e.s.d.'s involving l.s. planes.
Refinement. Refinement of *F*^2^ against ALL reflections. The weighted *R*-factor *wR* and goodness of fit *S* are based on *F*^2^, conventional *R*-factors *R* are based on *F*, with *F* set to zero for negative *F*^2^. The threshold expression of *F*^2^ > σ(*F*^2^) is used only for calculating *R*-factors(gt) *etc*. and is not relevant to the choice of reflections for refinement. *R*-factors based on *F*^2^ are statistically about twice as large as those based on *F*, and *R*- factors based on ALL data will be even larger.

### Fractional atomic coordinates and isotropic or equivalent isotropic displacement parameters (Å^2^)

**Table:**

	*x*	*y*	*z*	*U*_iso_*/*U*_eq_	
C1	0.5745 (2)	0.16484 (10)	1.05807 (18)	0.0509 (5)	
H1	0.6122	0.1859	0.9922	0.061*	
C2	0.4798 (3)	0.09802 (12)	1.0431 (2)	0.0657 (6)	
H2	0.4536	0.0743	0.9668	0.079*	
C3	0.4235 (3)	0.06598 (12)	1.1396 (2)	0.0694 (7)	
H3	0.3572	0.0217	1.1282	0.083*	
C4	0.4657 (3)	0.09960 (13)	1.2523 (2)	0.0649 (6)	
H4	0.4298	0.0773	1.3180	0.078*	
C5	0.5610 (2)	0.16633 (11)	1.26986 (19)	0.0522 (5)	
H5	0.5901	0.1884	1.3472	0.063*	
C6	0.61349 (19)	0.20050 (9)	1.17163 (16)	0.0419 (4)	
C7	0.7090 (2)	0.27484 (10)	1.19416 (16)	0.0430 (4)	
C8	0.6858 (2)	0.33474 (9)	1.09863 (16)	0.0411 (4)	
C9	0.5640 (2)	0.33689 (9)	0.99243 (17)	0.0430 (4)	
H9	0.4831	0.2992	0.9663	0.052*	
C10	0.7812 (2)	0.40322 (9)	1.10421 (16)	0.0432 (4)	
H10	0.8712	0.4169	1.1662	0.052*	
C11	0.71730 (19)	0.44609 (9)	1.00163 (15)	0.0397 (4)	
C12	0.7536 (2)	0.51834 (9)	0.95286 (17)	0.0426 (4)	
H12	0.8394	0.5488	0.9967	0.051*	
C13	0.4925 (2)	0.43088 (10)	0.82315 (17)	0.0459 (4)	
H13	0.4036	0.4015	0.7811	0.055*	
C14	0.5288 (2)	0.49875 (10)	0.77489 (16)	0.0454 (4)	
C15	0.6659 (2)	0.54404 (9)	0.84358 (16)	0.0432 (4)	
C16	0.4222 (3)	0.52534 (12)	0.6562 (2)	0.0625 (6)	
H16A	0.3335	0.4888	0.6298	0.094*	
H16B	0.4894	0.5290	0.5955	0.094*	
H16C	0.3756	0.5753	0.6672	0.094*	
C17	0.7185 (2)	0.61723 (11)	0.79141 (19)	0.0533 (5)	
C18	0.8515 (2)	0.73858 (11)	0.8309 (2)	0.0616 (6)	
H18A	0.7597	0.7674	0.7816	0.074*	
H18B	0.9315	0.7264	0.7807	0.074*	
C19	0.9329 (3)	0.78540 (12)	0.9384 (3)	0.0786 (7)	
H19A	0.8522	0.7980	0.9865	0.118*	
H19B	0.9765	0.8323	0.9110	0.118*	
H19C	1.0225	0.7561	0.9870	0.118*	
N1	0.58336 (16)	0.40372 (7)	0.93311 (12)	0.0394 (4)	
O1	0.80406 (17)	0.28578 (8)	1.29271 (13)	0.0604 (4)	
O2	0.7043 (3)	0.62853 (11)	0.68326 (16)	0.0966 (7)	
O3	0.78952 (16)	0.66744 (7)	0.87619 (13)	0.0533 (4)	

### Atomic displacement parameters (Å^2^)

**Table:**

	*U*^11^	*U*^22^	*U*^33^	*U*^12^	*U*^13^	*U*^23^
C1	0.0701 (11)	0.0331 (8)	0.0482 (11)	0.0025 (8)	0.0096 (8)	0.0030 (8)
C2	0.0881 (14)	0.0369 (9)	0.0660 (14)	−0.0047 (9)	0.0019 (11)	−0.0014 (9)
C3	0.0727 (13)	0.0413 (10)	0.0886 (18)	−0.0103 (9)	0.0041 (12)	0.0131 (11)
C4	0.0664 (12)	0.0542 (11)	0.0764 (15)	−0.0043 (9)	0.0202 (11)	0.0244 (11)
C5	0.0586 (10)	0.0493 (10)	0.0496 (11)	0.0033 (8)	0.0135 (8)	0.0081 (9)
C6	0.0465 (8)	0.0341 (8)	0.0451 (10)	0.0052 (6)	0.0094 (7)	0.0063 (7)
C7	0.0478 (8)	0.0384 (8)	0.0428 (10)	0.0027 (7)	0.0097 (7)	0.0009 (7)
C8	0.0484 (8)	0.0303 (8)	0.0447 (10)	0.0021 (6)	0.0097 (7)	−0.0028 (7)
C9	0.0485 (9)	0.0307 (8)	0.0493 (10)	−0.0017 (6)	0.0094 (7)	−0.0007 (7)
C10	0.0511 (9)	0.0346 (8)	0.0419 (10)	−0.0017 (7)	0.0049 (7)	−0.0040 (7)
C11	0.0457 (8)	0.0308 (8)	0.0424 (9)	0.0003 (6)	0.0088 (7)	−0.0056 (7)
C12	0.0506 (9)	0.0307 (8)	0.0469 (10)	−0.0021 (7)	0.0115 (7)	−0.0046 (7)
C13	0.0497 (9)	0.0399 (9)	0.0450 (10)	0.0019 (7)	0.0026 (7)	−0.0038 (8)
C14	0.0544 (9)	0.0392 (9)	0.0429 (10)	0.0078 (7)	0.0105 (7)	−0.0032 (7)
C15	0.0534 (9)	0.0329 (8)	0.0460 (10)	0.0048 (7)	0.0163 (7)	−0.0016 (7)
C16	0.0757 (13)	0.0548 (11)	0.0511 (12)	0.0037 (10)	−0.0005 (10)	0.0053 (10)
C17	0.0625 (11)	0.0446 (10)	0.0538 (12)	0.0022 (8)	0.0146 (9)	0.0094 (9)
C18	0.0614 (11)	0.0425 (10)	0.0830 (16)	−0.0018 (8)	0.0202 (10)	0.0214 (10)
C19	0.0987 (16)	0.0453 (11)	0.0980 (19)	−0.0186 (12)	0.0344 (14)	0.0007 (12)
N1	0.0462 (7)	0.0303 (7)	0.0412 (8)	0.0017 (5)	0.0077 (6)	−0.0027 (6)
O1	0.0713 (8)	0.0561 (8)	0.0481 (8)	−0.0085 (6)	−0.0003 (6)	0.0048 (6)
O2	0.1448 (16)	0.0845 (13)	0.0577 (11)	−0.0378 (12)	0.0149 (10)	0.0161 (10)
O3	0.0681 (8)	0.0339 (6)	0.0612 (9)	−0.0057 (5)	0.0208 (6)	0.0044 (6)

### Geometric parameters (Å, º)

**Table:**

C1—C2	1.379 (3)	C11—C12	1.416 (2)
C1—C6	1.388 (3)	C12—C15	1.356 (2)
C1—H1	0.9300	C12—H12	0.9300
C2—C3	1.375 (3)	C13—C14	1.348 (3)
C2—H2	0.9300	C13—N1	1.380 (2)
C3—C4	1.365 (4)	C13—H13	0.9300
C3—H3	0.9300	C14—C15	1.448 (2)
C4—C5	1.381 (3)	C14—C16	1.500 (3)
C4—H4	0.9300	C15—C17	1.491 (2)
C5—C6	1.392 (3)	C16—H16A	0.9600
C5—H5	0.9300	C16—H16B	0.9600
C6—C7	1.495 (2)	C16—H16C	0.9600
C7—O1	1.224 (2)	C17—O2	1.207 (3)
C7—C8	1.470 (2)	C17—O3	1.326 (2)
C8—C9	1.382 (2)	C18—O3	1.458 (2)
C8—C10	1.409 (2)	C18—C19	1.485 (3)
C9—N1	1.356 (2)	C18—H18A	0.9700
C9—H9	0.9300	C18—H18B	0.9700
C10—C11	1.373 (2)	C19—H19A	0.9600
C10—H10	0.9300	C19—H19B	0.9600
C11—N1	1.404 (2)	C19—H19C	0.9600
			
C2—C1—C6	119.8 (2)	C11—C12—H12	119.3
C2—C1—H1	120.1	C14—C13—N1	121.98 (15)
C6—C1—H1	120.1	C14—C13—H13	119.0
C3—C2—C1	120.8 (2)	N1—C13—H13	119.0
C3—C2—H2	119.6	C13—C14—C15	117.81 (16)
C1—C2—H2	119.6	C13—C14—C16	118.96 (16)
C4—C3—C2	119.6 (2)	C15—C14—C16	123.17 (16)
C4—C3—H3	120.2	C12—C15—C14	120.47 (16)
C2—C3—H3	120.2	C12—C15—C17	119.22 (16)
C3—C4—C5	120.8 (2)	C14—C15—C17	120.21 (16)
C3—C4—H4	119.6	C14—C16—H16A	109.5
C5—C4—H4	119.6	C14—C16—H16B	109.5
C4—C5—C6	119.9 (2)	H16A—C16—H16B	109.5
C4—C5—H5	120.1	C14—C16—H16C	109.5
C6—C5—H5	120.1	H16A—C16—H16C	109.5
C1—C6—C5	119.08 (17)	H16B—C16—H16C	109.5
C1—C6—C7	123.12 (16)	O2—C17—O3	123.24 (18)
C5—C6—C7	117.79 (16)	O2—C17—C15	123.64 (19)
O1—C7—C8	120.54 (16)	O3—C17—C15	113.05 (17)
O1—C7—C6	119.69 (16)	O3—C18—C19	107.76 (18)
C8—C7—C6	119.76 (14)	O3—C18—H18A	110.2
C9—C8—C10	107.95 (15)	C19—C18—H18A	110.2
C9—C8—C7	127.19 (15)	O3—C18—H18B	110.2
C10—C8—C7	124.77 (15)	C19—C18—H18B	110.2
N1—C9—C8	107.87 (14)	H18A—C18—H18B	108.5
N1—C9—H9	126.1	C18—C19—H19A	109.5
C8—C9—H9	126.1	C18—C19—H19B	109.5
C11—C10—C8	107.68 (14)	H19A—C19—H19B	109.5
C11—C10—H10	126.2	C18—C19—H19C	109.5
C8—C10—H10	126.2	H19A—C19—H19C	109.5
C10—C11—N1	107.06 (14)	H19B—C19—H19C	109.5
C10—C11—C12	136.21 (15)	C9—N1—C13	128.99 (14)
N1—C11—C12	116.74 (14)	C9—N1—C11	109.44 (14)
C15—C12—C11	121.40 (15)	C13—N1—C11	121.57 (14)
C15—C12—H12	119.3	C17—O3—C18	115.64 (17)
